# Investigating the Usability and Acute Effects of a Bedside Video Console to Prefrontal Cortical Activity Alterations: A Preclinical Study in Healthy Elderly

**DOI:** 10.3389/fnsys.2017.00085

**Published:** 2017-11-28

**Authors:** Ruud H. Knols, Jaap Swanenburg, Dino De Bon, Federico Gennaro, Martin Wolf, Bernard Krüger, Dominique Bettex, Eling D. de Bruin

**Affiliations:** ^1^Directorate of Research and Education, Physiotherapy & Occupational Therapy Research Center, University Hospital Zurich, Zurich, Switzerland; ^2^Department of Chiropractic Medicine, Faculty of Medicine, Balgrist University Hospital, University of Zürich, Zurich, Switzerland; ^3^Department of Health Sciences and Technology, Institute of Human Movement Sciences and Sport, ETH Zurich, Zurich, Switzerland; ^4^Biomedical Optics Research Laboratory, Division of Neonatology, University Hospital Zurich, Zurich, Switzerland; ^5^Institute of Anesthesiology, University Hospital Zurich, Zurich, Switzerland

**Keywords:** brain plasticity, computerized cognitive training, cortex, elderly, exergames, motor control, multisensory integration, neurorehabilitation

## Abstract

Elderly people at risk of developing cognitive decline; e.g., following surgery, may benefit from structured, challenging, and repetitive cognitive video training. This study assessed usability and acute effects of a newly developed bedside console (COPHYCON). Fifteen healthy elderly individuals performed a one-time 80-min intervention, including cognitive video games aimed at improving awareness and selective attention. Perceived usefulness and perceived ease of use (Technology Acceptance Model) were assessed together with measures of the achieved game level, reaction times, (in-) correct responses during ALERT and SELECT game play. Further, prefrontal cortical involvement of the regional cerebral hemoglobin saturation (rS02%) assessed with functional near infrared spectroscopy (fNIRS) (*n* = 5) and EEG power (*n* = 10) was analyzed. All participants completed the study without any adverse events. Perceived usefulness and perceived ease of use (TAM scores range 1–7) of the system varied between 3.9 and 6.3. The game levels reached for awareness varied between 9 and 11 (initial score 8–10), for reaction speed between 439 and 469 ms, and for correct responses between 74.1 and 78.8%. The highest level for the selective attention games was 2 (initial score 1), where reaction speed varied between 439 and 469 ms, correct responses between 96.2 and 98.5%, respectively. The decrease of rS02% in the right prefrontal cortex during gameplay was significantly (*p* < 0.001) lower, compared to the left prefrontal cortex. Four participants yielded significant lower rS02% measures after exergaming with the ALERT games (*p* < 0.000), but not with the SELECT games. EEG recordings of theta power significantly decreased in the averaged ~0.25–0.75 time interval for the left prefrontal cortex sensor across the cognitive game levels between the ALERT 1 and SELECT 1, as well as between SELECT 1 and 2 games. Participants rated the usability of the COPHYCON training positively. Further results indicate that video gaming may be an effective measure to affect prefrontal cortical functioning in elderly. The results warrant a clinical explorative study investigating the feasibility of the COPHYCON in a clinical setting.

## Introduction

The early twenty-first century confronts us with a dilemma concerning old age, as there is expected to be an unprecedented increase in the numbers of elderly people (Esiri and Chance, 2012). This is relevant because, e.g., the human brain experiences a variety of prominent structural changes during the course of aging (Deary et al., 2009; Scafato et al., 2010; Eggenberger et al., 2016), which often leads to a functional decline and disability (Borson, 2010). Structurally, the brain's size decreases with anterior to posterior progression, leaving the prefrontal regions most affected by this aging process. Increased age is also associated with a reduction of cognitive functioning, which is prevalent in almost every second elderly person (Scafato et al., 2010). Although the evidence appears not to be sufficient to conclude that modified brain structures reflect the neuroanatomical substrates for the age-related decline of cognitive performance (Fjell and Walhovd, 2010; Salthouse, 2011; Bennett and Madden, 2014; Eggenberger et al., 2016). This seeming discrepancy might be explained due to the ability of the aging brain to compensate for structural atrophy by amending recruitment patterns that support functioning and maintain cognitive functions (Deary et al., 2009; Grady, 2012; Eggenberger et al., 2016). Deterioration of the mental learning processes may affect a variety of cognitive domains, such as psycho-motor skills, (working-) memory, and executive functions (Skvarc et al., 2016). Executive functions are “higher-order aspects of cognition that are strongly dependent upon the integrity of the prefrontal cortex” (Lipnicki et al., 2009).

Working memory, being a vital process underlying human thought, is necessary for everyday decision-making and problem solving, making it a fundamental process in the daily lives of older adults (Nissim et al., 2016). It relies on frontal lobe structures and is known to decline with age (Nissim et al., 2016). Activities of daily living as organizing and planning daily routines and appointments require working memory and other components of the executive functions (Mograbi et al., 2014; Nissim et al., 2016). Thus, a decline in working memory capacity may lead to loss of independence and decreased quality of life (Klingberg, 2010).

Furthermore, cognitive decline may appear after surgery in 25% of the elderly population. Oxidative stress and adjacent neuro-inflammation of the brain are associated with cognitive decline, e.g., in post-operative delirium (Skvarc et al., 2016), however, the mechanisms of cognitive decline following surgery cannot be explained exactly for all elderly. The burden of post-operative cognitive deterioration is arduous, given the increased length of hospital sojourning, including prolonged stay on intensive care unit(s), and reduced functional performance; factors that not only have strong effects on the subjective way patients perceive the quality of their life, but also have an impact on the healthcare systems by increasing costs of care (Cropsey et al., 2015).

Cognitive training may reduce the risk of cognitive decline in elderly (Baumgart et al., 2015). A potential countermeasure to impede the cognitive decline is aerobic exercise-, as research showed that participation in aerobic exercise interventions improves functioning of the prefrontal cortex and performance of executive functioning tasks (Lipnicki et al., 2009). Improvement of the corticospinal excitability, a neurophysiologic mechanism related to muscle functioning, may also ameliorate the activity of the prefrontal cortex and executive functions of the brain (Kawakami et al., 2001). In a recent study with male adolescents, Benzing et al. reported that higher order cognition was immediately enhanced by cognitively engaging exergame-based physical activity (PA). A PA condition with a high level of cognitive engagement resulted in significantly better performance in cognitive flexibility compared to conditions with low levels of cognitive engagement (Benzing et al., 2016). Exergames are “technology-driven physical activities, such as, videogame play, that require participants to be physically active or exercise in order to play the game^1^.” Both aerobic exercise and cognitive training in elderly participants are reported to improve brain health of the trainees, however, specificity of training seems to apply (Chapman et al., 2016). Chapman et al. (2016) showed that exercisers with greater gains in memory benefitted from higher central blood flow in the hippocampal regions, a brain area particularly vulnerable regarding aging related deterioration (e.g., dementia). These findings were consistent with a report of Shatil et al. (Shatil, 2013) where the authors proposed that cognitive training may be the main treatment for cognitive gains in elderly when compared to the benefit of isolated physical exercise.

More recent approaches showed that cognitive functioning may benefit from simultaneous cognitive-physical exercise programs, where traditional exercise components; e.g., aerobic and/or resistance training, are combined with video-game based cognitive training (Eggenberger et al., 2015). It can be hypothesized that a cognitive-physical training program delivered through exergames in elderly threatened with cognitive decline after medical treatment (e.g., surgery), shows effect on cognitive functioning (de Bruin et al., 2010). This is important since these elderly individuals have an impaired capacity to train adequately with aerobic types of training due to surgery related immobilization. Additionally, recent studies identified the potential for cognitive (motor-) training as a promising form of exercise for immobilized elderly patients in acute care settings (Pichierri et al., 2011; Schoene et al., 2014; van het Reve and de Bruin, 2014).

**Figure 1:**
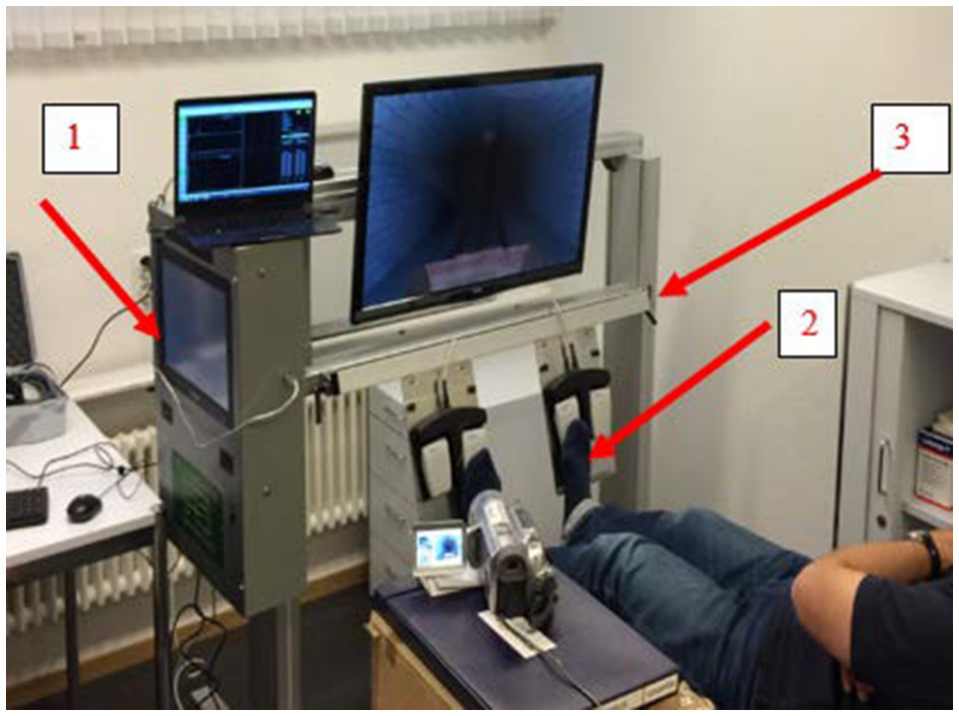
Patient Bed Side Cognitive Gaming Console (COPHYCON-Prototype) in action while performing COGNIPLUS games during EEG measures. The exergames are stored in an all in one computer (1) and operated by foot pedals (2). With the lifting column (3), the height of the device is installed above a patient's bed. The participant in this figure gave written consent for publication.

In accordance with the phased iterative approach suggested by Campbell et al. (2000) we designed and evaluated a bedside COGnitive and PHYsical training CONsole (COPHYCON) (Figure 1) for use in acute care patients (e.g., on intermediate and intensive care units). Before larger scale studies and application in clinical practice are to be considered, possible new treatments usually have to go through a series of phases to test whether they are safe and effective (Thabane et al., 2010; Bower et al., 2015). We, therefore, performed a trial including a single session of exergaming based on the model for complex interventions advocated by the British Medical Research Council (Craig et al., 2008). This was performed to test the usability and acute effects of the COPHYCON (Esiri and Chance, 2012) in terms of acceptance, adherence, and attrition (Lipnicki et al., 2009) and the acute effect of a COPHYCON exercise session on measures of brain functioning in a group of untrained elderly.

We hypothesized, that video game-play with the COPHYCON is usable and affects prefrontal cortical involvement.

## Materials and methods

### Study design

Usability was assessed with a user-centered interaction design. The study was approved by the Ethics Committee (PB_2016-00458) of the canton of Zurich and the Swiss Agency for Therapeutic products (SWISSMEDIC, Berne, Switzerland); (EUDAMED CIV-15-01-013107). All participants gave their written consent prior to study entry. The ClinicalTrials.gov Identifier number is NCT02661230.

### Participants

Between December 2015 and January 2016, usability and effects were assessed in a convenience sample of 15 (*n* = 15) healthy elderly recruited from the greater area of Zurich. Participants were included if they lived independently, were older than 55 years of age and had a MOCA score of at least 25 points (Tan et al., 2015). Individuals with mild cognitive impairment or dementia, acute or chronic health problems requiring medical treatment by a physician, and/or currently participating in another similar study were excluded.

### *COPHYCON* prototype

A prototype of the *COPHYCON* was designed and constructed by mechanical and electrical engineers of the Paul Scherrer Institute, Villigen, Switzerland. The aluminum construction is a mobile device on rolls carrying an all-in-one-computer, a LCD-TV screen and pressure sensitive pads where the hands or feet can be placed to enable user interaction with the video games. The *COPHYCON* can be easily positioned over and removed from the bed of the patient (Figure 1). The participants were required to control the device in supine position, while pressing sensors with their feet. The weight of the device is ~80 kg (176 lbs), the length is 124 cm and the height 90 cm in retracted and 150 cm in maximum extended position, respectively. The prototype was built for scientific purposes. Before using the device in the study, it passed the electromagnetic comparability (EMC) and Security requirements performed by ELEKTROSUISSE in June 2015 (Zurich and Fehraltorf, Switzerland). All procedures and documents were checked by Swissmedic in advance of the study. (Severe-) adverse events and device related events were not expected. However, safety issues for the participants were evaluated since this prototype currently holds no EU conformity sign.

### Video games procedures

All participants performed a single training session with “CogniPlus” (SCHUHFRIED, (http://www.schuhfried.at; van het Reve and de Bruin, 2014), which was uploaded on the all-in-one-computer of the COPHYCON. During the experiment, participants were positioned in a comfortable supine position in a dimly lit environment.

In the ALERT training program, the ability to increase and maintain attention intensity in the short term is trained.

### Scene and task of the alert training program

A motorcycle drives along a winding road. The task of the participant is to observe the road section carefully and to press the key with the foot as quickly as possible when obstacles occur. If the reaction time is adequate, the motorcycle decelerates/stops and continues its journey after the obstacle has disappeared. In the case of a delayed reaction, “emergency braking” occurs: The sound of brakes is heard, the motorcycle stops and a yellow exclamation mark is displayed on the screen.

If the attention intensity is exogenously activated by a sudden warning signal, then this is defined as phasic alertness. If patients are activated to press the key without a warning, then this is referred to as intrinsic alertness. The aim of an alertness training must be to increase the intrinsic alertness, since the activation is exclusively cognitively controlled. In case of deficits of alertness, however, it is necessary in a first step to improve the phasic alertness and only then begin training of intrinsic alertness.

#### Training modes

The training program ALERT comprises two training modes: The AS1 mode exercises phasic alertness, the S2 mode intrinsic alertness. In the training form AS1 the obstacles for the external activation of attention are announced acoustically and visually by warning signals. In training form AS2, the acoustic and visual alarms are eliminated. The motorcycle now drives through a nightly nebulous landscape where the obstacles suddenly appear from the mist.

#### Levels of difficulty

Both S1 and S2 training forms have 18 levels of difficulty. The requirement increases by shortening the maximum permissible reaction time. While the user still has 1.8 s in the first stage to react to an obstacle, only 0.3 s remain between the sudden appearance of an obstacle and the emergency braking in the last stage. In the first session, the client is classified according to the speed of his first reactions into a performance-adequate difficulty level. This ensures that the training program adapts optimally to the participant's performance at the beginning of the training.

The training program SELECT is used to improve the ability to respond quickly to relevant stimuli and suppress inappropriate reactions.

### Scene and task of the select training program

The user drives through a tunnel in a mine hutch. Relevant and irrelevant stimuli (optical, acoustic, or crossmodal) suddenly appear from the dark. The client should only react to relevant stimuli. In the case of a delayed or omitted response to a relevant stimulus, a negative feedback in the form of thunder and lightning is given. If the client falsely reacts to an irrelevant stimulus, the figure or sound source is illuminated in red.

This training program for selective attention intends to make it easier for the participant to distinguish quickly between relevant and irrelevant aspects of a task. Tasks for selective attention usually require a rapid decision within a stimulus set, in which the relevant and irrelevant stimuli are clearly defined.

#### Training forms

The training program SELECT comprises two training forms: (1) The training form SEL1 trains selective attention in the visual modality. (2) In acoustic training form SEL2, it is the task of the client to selectively react to relevant noise. The training module SEL3, in which the client is instructed to react to certain stimulus combinations, was not applied in this study.

#### Levels of difficulty

There are 15 difficulty levels for every training form. SELECT works with a two-fold adaptation to the participants' performance level. On the one hand, the number of relevant and irrelevant stimuli decreases or increases. On the other hand, the maximum time available for a timely response adapts to the client's reaction level over all difficulty levels. For a high-performance client the pit truck becomes faster after the first reactions have been registered. This ensures that the training program adapts optimally to each client's performance at the beginning of the training.

The sequence of order of the exergame training session was: Phasic (AS1) and Intrinsic Alertness (AS2) (Hauke et al., 2011; Hershey, 2014). Then, the selective attention (van het Reve and de Bruin, 2014) in the visual modality (SEL1) and the acoustic training form (SEL2) (Table 1).

**Table 1:** Demographics and clinical characteristics of participants (*n* = 15).

	**Mean**	**SD**	**Median**	**Range**
Age (years)	63.7	5.4	63	56–75
Height (cm)	173	7.8	173	160–186
Weight (kg)	74.6	17.9	70	50–122
BMI (kg/m^2^)	24.8	4.8	23.6	17.5–36
MoCa	28	0.83	28	27–30
**Computer-use** **Game-use**	**n**	**%**	**n**	**%**
Daily	12	80	1	6.66
Weekly	2	13.33	6	40
Monthly	1	6.66	1	6.66
Never	–	–	7	46.66
Education level	n	%	–	–
Secondary school	–	–	–	–
Vocational education	5	33.33		
Higher professional education	7	46.66		
College/University	3	20		

### Game protocols

**Figure 2:**
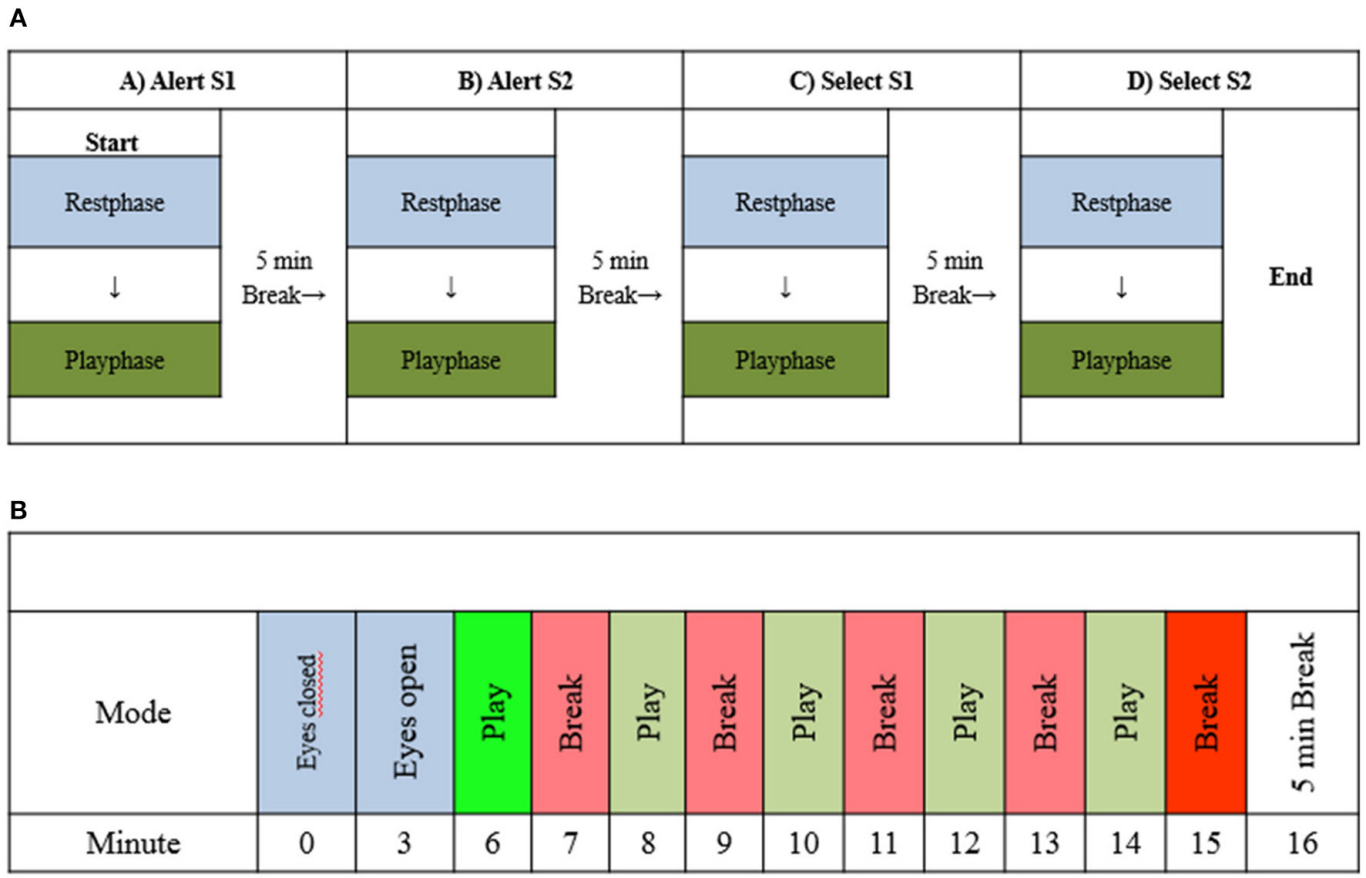
**(A)** Sequence of the exergaming protocol. The Intervention consisted out of four exergames: “Alert S1, Alert S2, Select S1 and Select S2”, consisting in a rest (blue) and game phase (green). A 5 min pause was provided between the games. **(B)** Every game sequence lasted approximately 21 min: A focus phase of 2 × 3 min (blue; 0 and 3), 5 × 1 gaming phase (green; 6, 8, 10, 12, 14), 5 × 1 min break phase (red; 7, 9, 11, 13, 15) and a 5 min recovery phase (white;16). This sequence was repeated four times.

All measurements were performed at the Physiotherapy Department of the University Hospital Zurich. During the experiment, participants were positioned in a comfortable supine position in a dimly lit environment. Prior to every game phase, the participant had to concentrate for 6 min (3 min eyes open fixing on a point on the TV-screen and 3 min eyes closed). Then the game level was determined for every participant. The game sequence for every participant was: (1) Phasic Alert S1(AS1), (2) Intrinsic Alert S2 (AS2), (3) Phasic select S1 (SEL1) and (4) Intrinsic Alert S2 (SEL2), receiving 5 × 1 min of gaming exercise, following a 1 min rest interval after every game interval. After every completed game phase of 5 × 1 min, the participant received a break for 5 min (Figure 2). The game protocol was identical for the participants randomized to fNIRS and EEG measures.

### Outcomes

#### Primary usability Outcome(S)

Perceived usefulness and perceived ease of use of the training technology, which are the main components of technology acceptance behaviors, were evaluated with an abridged version of the technology acceptance model questionnaire which evaluated participants' perceived acceptance of the intervention at post-exercise session (de Graaf et al., 2013) (TAM Davis, 1989). Responses were recorded using a 7-point Likert scale ranging from “strongly disagree” (rated as 1) to “strongly agree” (rated as 7). The main objective of the model is to provide a foundation for determining the impact of external factors on internal beliefs of attitude and intention (Jokar et al., 2017). The TAM model can explain the users' motivation by three factors: perceived ease of use (which shows the extent to which a user expects the use of the system needs effort), perceived usefulness (the extent to which an individual believes the use of a certain system will improve performance, Taylor and Todd, 1995), and attitude toward using the technology (Davis, 1989).

The TAM explains the variance in attitude toward a technology and determines a comparable percentage of variance in usage (Porter, 2006; Jokar et al., 2017).

For attrition, the number of participants lost during the intervention was recorded. For adherence, participants' engagement with the four games was assessed. Adherence was calculated as the number of completed game sessions as a percentage of the maximal possible game plays. Game play metrics was assessed while exergaming was actively performed. The *Intensity Level (Alert level 1 to level 18/Select level 1 to level 15*), the maximum achieved cognitive game level, the type of cognitive gaming (Alert S1 = AS1, Alert S2 = AS2, Select S1 = SEL1, Select S = SEL2), reaction speed during cognitive gaming (ms), the amount (in-)correct reactions during cognitive gaming (%), and false positive reactions (%) were registered.

#### Safety

The participants were continuously monitored for the occurrence of (Severe) Adverse Events (SAE) and health hazards during and immediately after the training sessions. The device was checked for malfunctioning.

### Secondary outcomes

The 15 participants were randomly allocated into two subsamples, either Functional Near Infrared Spectroscopy (fNIRS, *n* = 5) or Electroencephalography (EEG, *n* = 10). Both recordings were performed on the prefrontal cortex (PFC) area.

Randomization was accomplished by use of a computerized random table.

fNIRS measures the increase in Intracranial regional hemoglobin oxygen saturation (Ferrari et al., 2004; Denault et al., 2007; Covidien, 2018). The *INVOS OXYMETER 5100C (Covidien Somanetics, Troy, MI 48084, USA)* device was used to measure absolute values of the regional cerebral hemoglobin saturation (rSO2) in the left and right PFC during exergaming. Self-adhesive optodes placed on both sides of the forehead dispense a modulated light beam at 730 and 810 nm distinct wavelengths through the underlying brain substance^2^. The area of measurement is defined by the brain tissue under the optode and allows high spatial resolution^2^.

The baseline rSO2 level was individually determined during 5 min before starting the intervention (Baseline Game rSO2 level). For the Alertness (AS1, AS2) and the selective attention (SEL1 and SEL2) game conditions, the average level of rSO2 from the 60 s that preceded active game play were subtracted from the observed levels of rSO2 while actively playing the game. fNIRS data was recorded continuously at 10 Hz and measurement values were shown online on the INVOS display. The two self-adhesive NIRS optodes (Somasensor® SAFB-SM, Covidien, MA, USA) were placed on the participants' forehead, according to the international 10/20 EEG electrode placement system (Jasper, 1958), covering the cortical areas between Fp1-Fp3-Fp7 and Fp2-Fp4-Fp8. These areas, respectively, correspond to the left and the right PFC in a supine lying participant (Kim et al., 2000; Matsuda and Hiraki, 2006; Leff et al., 2008). The time-points between active gameplay and a break phase, or vice versa, were marked by the investigator in the INVOS™ system. All data were analyzed with the INVOS Cerebral Somatic Oximeter Analytics program (Covidien, 2018).

EEG activity was recorded from two Ag/AgCl sensors (Ambu® White Sensors, Ambu A/S, Denmark) over the left and right PFC (Fp1 and Fp2) according the international 10/20 electrode placement system and sampled at 128 Hz (Alpha-Active Ltd, Devon, UK). As recording reference and ground, a passive common mode sense (CMS) electrode and two active driven right leg (DRL) electrodes were placed at Fpz and on the left/right mastoids, respectively. Before electrode placement, the skin was prepared and cleaned with an abrasive skin preparation gel (NuPrep®, Weaver and Company, Aurora, CO, USA). EEG data analysis was performed using custom scripts written in MATLAB R2017a (The Mathworks, Natick, MA) including functions from both EEGLAB 14.1.0b (Delorme and Makeig, 2004), ERPLAB (Lopez-Calderon and Luck, 2014) and Fieldtrip-20170517 (Oostenveld et al., 2011) open source toolboxes. EEG data were high-pass filtered [zero-phase Hamming windowed sinc FIR, cut-off frequeny (−6 dB) 0.5 Hz, passband edge 1 Hz, transition bandwidth 1 Hz, order 425] and subsequently low-pass filtered [zero-phase Hamming windowed sinc FIR, cut-off frequeny (−6 dB] 33.75 Hz, passband edge 30 Hz, transition bandwidth 7.5 Hz, order 59]. EEG data were then segmented in 2-s epochs time locked around specific stimuli (i.e., reacting on the onset of obstacles before an approaching motorcycle or figures popping-up in front of a moving mine hutch) from the cognitive game level (0.75 s pre and 1.25 post stimulus onset). The amount of stimuli, and therefore of those epochs, were set as variable with respect to the cognitive game level and between subjects. On average, for all the four cognitive game levels (AS1, AS2, SEL1, SEL2), the number of collected epochs was ~116 (SD: ±24; range: 83–155). EEG epochs containing artifacts (e.g., ocular movements and eye blinks) were automatically rejected using a sliding window (width: 200; step: 100 ms) with an absolute peak-to-peak voltage threshold criterion of ± 100 μV. The remaining epochs exceeding both locally and globally the average activity probability by ± 5 SD were also rejected. On average, ~83 total epochs remained for further analysis (~73% of each participant; SD: ~11%; range: ~59–89%). Only stimuli for actual cognitive game playing were considered, however, except those originating from preparatory phases of the respective level. Furthermore, in the rest phases between actively playing the games, two periods of background EEG were recorded (eyes open and eyes closed, respectively) and segmented into epochs of 2 s for further analysis. Epochs from these EEG data periods were screened automatically for artifact detection and rejection using the same criteria from above.

EEG data and stimuli were video-synchronized with simultaneous recording of gameplay and EEG-screen (Sony HDR XR520VE, HD resolution 1,920 × 1,080 at 16 mbits). The recorded video was visually inspected frame-by-frame using appropriate software (Avidemux 2.6) to identify the game stimuli onsets and align these with the EEG data.

### Data analysis

All data were stored in Microsoft Excel 2010. IBM SPSS version 23.0 was used for data analysis. Normality of the data was evaluated using the Kolmogorov Smirnov test for the fNIRS data. The study population, clinical characteristics and usability outcomes (implementation, acceptance, and safety) were defined adopting descriptive statistics (Tables 1, 2; Norman and Streiner, 2008).

**Table 2:** Usability results for Cognitive gaming Alert S1 (AS1) and S2 (AS2) and Select S1 (SEL1) and SEL2 (ACS2).

**Game type**		**AS1**	**AS2**	**SEL 1**	**SEL2**
Initial game level after game test.	Mean	8	10	1	1
Alert 1–18	SD	1.19	1.71	–	–
Select 1–15	Range	7–10	7–13	–	–
Highest	Mean	9	11	2	2
Game-Level	SD	1.19	11.71	0.35	0.41
Alert 1–18	Range	8–11	8–14	1–2	1–2
Reaction speed (ms)	Mean	574	488.27	439.07	469.60
Select 1–15	SD	57.81	65.28	34.89	44.36
Correct responses (%)	Mean	78.80	74.07	98.47	96.20
	SD	10.02	9.97	3.10	3.45
Falls positive reactions (%)	Mean	n.a.	n.a.	6.13	5.80
	SD	n.a.	n.a.	5.01	4.21

fNIRS data were analyzed with a *t*-test or the Wilcoxon signed-rank test (Table 3), the Pearson and Spearman correlation test, as well as linear regression analyses where appropriate (Table 4).

**Table 3:** Intracranial regional hemoglobin oxygen saturation (rS02) (mean, median, and SD) values and significant differences between the left and right prefrontal cortical areas.

	**Phase**		**Left prefrontal lobe**	**Right prefrontal lobe**	***P* < 0.05**
1	Introduction followed by concentration phase (eyes open/eyes closed)	Mean	67.17	67.27	0.223^ND^
		Median	67.20	67.30	
		SD	1.25	1.17	
2	Alert AS1 game fase	Mean	67.54	67.43	0.220
		Median	67.50	67.40	
		SD	0.44	0.34	
3	Break 1 followed by concentration phase (eyes open/eyes closed)	Mean	67.06	66.68	<0.001^ND^ ^ES = 0.^
		Median	67.00	66.60	
		SD	0.47	0.44	
4	Alert AS2 game fase	Mean	66.94	66.69	0.010 ^ES = 0.5^
		Median	66.90	66.80	
		SD	0.38	0.58	
5	Break 2 followed by concentration phase (eyes open/eyes closed)	Mean	66.35	65.90	<0.001 ^ES = 0.7^
		Median	66.40	66	
		SD	0.38	0.43	
6	SELECT SEL1 game fase	Mean	66.60	66.48	0.095^ND^
		Median	66.67	66.60	
		SD	0.33	0.38	
7	Break 3 followed by concentration phase (eyes open/eyes closed)	Mean	66.18	65.88	0.005 ^ES = 0.5^
		Median	66.20	65.80	
		SD	0.44	0.57	
8	SELECT SEL1 game fase and break 4	Mean	66.14	65.92	0.002 ^ES = 0.5^
		Median	66.00	65.80	
		SD	0.47	0.46	
	Total phase 1–8	Mean	66.75	66.54	<0.001 ^ES = 1.1^
		Median	66.79	66.60	
		SD	0.77	0.84	
			rS02 decrease	rS02 decrease	
			*p* < 0.000	*p* < 0.000	

*ND, Normal distribution of the data: Data were normally distributed and analyzed with a t-test for paired samples. All non-normally distributed data were analyzed with a Wilcoxon signed-rank. ES, Cohends dz Effect size*.

**Table 4:** Results for individual participant regression analyses of the left and right prefrontal cortex during exergaming (A1, A2, SEL1, SEL2).

**Participant**	**AS1**	**Slope/Intercept rS02**	**AS2**	**Slope/Intercept rS02**	**SEL1**	**Slope/Intercept rS02**	**SEL2**	**Slope/Intercept rS02**
1 Left	0.000^*^	74.2/−0.17	0.045	71.8/−0.06	0.262	70.2/−0.023	0.448	65.2/0.035
1 Right	0.001^*^	68/−0.84	0.047	68.3/−0.06	0.005	69.3/−0.06	0.651	66.3/−0.02
2 Left	0.004	71.7/−0.07	0.200	71.9/−0.045	0.262	67.2/0.032	0.175	73.1–0.054
2 Right	0.000^*^	73.3/−0.09	0.003	73.6/−0.074	0.167	67.9/0.033	0.690	70.8/−0.014
3 Left	0.093	−72.5/−0.044	0.984	71.4/−0.001	0.134	69.7/0.041	0.09	68.9/0.042
3 Right	0.046	67.7/0.057	0.708	69.7/−0.009	0.889	69.2/0.003	0.049	65.1/0.052
4 Left	0.000^*^	65.5/−0.113	0.266	63.4/−0.027	0.080	59.3 /0.037	0.042	54.5/0.090
4 Right	0.000^*^	68.5/−0.155	0.037	65.9/−0.050	0.180	61.3/0.040	0.120	57.2/0.083
5 Left	0.640	60.0/−0.007	0.000^*^	63.4/−0.096	0.636	59.9/−0.010	0.466	60.6/−0.025
5 Right	0.249	64.1/0.021	0.000^*^	67.4/−0.080	0.804	63.8/0.005	0.353	64.9/−0.024

Significant p-values

* after Bonferroni correction (p < 0.001)^*^.

The preprocessed EEG data were then transformed and subsequently analyzed in either time-frequency domain (i.e., EEG event-related data epochs) or in the frequency domain (EEG background at rest). In the first case, from all the EEG epochs, participants and game level (i.e., AS1, AS2, SEL1, and SEL2) Time-Frequency Representations (TFRs) were calculated using a single Hanning taper within the entire time length of the epochs in a priori selected frequency bands of interest (FOI, i.e., Theta and Alpha) with a spectral resolution of 2 Hz. For each of these FOI a specific frequency was chosen (5.5 and 10.5 Hz, respectively) with a fixed spectral smoothing of ± 2 Hz, therefore encompassing the entire FOI (i.e., 3.5–7.5 and 8.5–12.5, for Theta and Alpha FOI respectively) without overlapping. De-meaning as well as de-trending of the EEG data segments was performed before the TFRs computations. In the case of EEG background at rest, the frequency power of the EEG data segments was calculated adopting the same parameters as described above but employing the Fast Fourier Transform (FFT). Given that the power spectra obtained from both the TFRs and FFT calculations are by definition not normally distributed, they were then log-transformed. However, this procedure was also performed in order to increase the sensitivity of the subsequent statistical testing. In order to compare both TFRs and FFT power spectra across the game levels, a Multivariate Analysis of Variance (MANOVA) F-statistic with non-parametric permutation test was employed. Probability (*p*-values) was approximated by 1,000 Monte Carlo permutations and the level of statistical significance was set to *p* < 0.05. In order to increase the sensitivity of the statistical testing, the time dimension of the TFRs were partitioned into three averaged post-stimulus onset time bins (~0–0.25 s, ~0.25–0.5 s, ~0.5–0.75 s). In both TFRs and FFT power spectra statistical comparisons, Theta and Alpha FOI were chosen *a priori* because of their known involvement in exergaming and aging (Mathewson et al., 2012; Rossini et al., 2013; van der Kooi et al., 2015; Schättin et al., 2016). Multiple comparisons were corrected by False Discovery Rate (FDR) and, given that MANOVA is an omnibus statistical test, *post-hoc* analyses were carried out for selected partial comparisons using a two-tailed dependent *t*-test with non-parametric Monte Carlo permutations (1,000 permutations with *p* < 0.05). All the statistics in respect to the EEG data analysis were performed with Fieldtrip-20170517 (Oostenveld et al., 2011).

Effect size estimates (Cohens d_z_) were calculated for fNIRS (Table 3) and EEG data (Lakens, 2013).

## Results

Fifteen participants were included in this study (five women, 10 men) aged 63.7 (SD 5.4) years. Four men and one woman were randomized to NIRS measurements and four women and six men were randomized to EEG measurements, respectively. The demographic and baseline characteristics of the participants are reported in Table 1.

### Primary usability outcomes

For the TAM Questionnaire Items, the mean value for “perceived ease of use” was 6.3 (SD 0.2, range 4–7), the mean value for “perceived usefulness” was 5.7 (SD 0.4, range 2–7), the mean value for “attitude toward using” was 3.9 (SD 2.5, range 1–7) and the mean value for “behavioral intention to use” was 4.9 (SD 0.3, range 1–7). All 15 participants completed the gaming protocol (Attrition 0% and adherence 100%) including the four games AS1, AS2, SEL1, and SEL2 in 79 min, rendering an adherence of 100%. The game levels achieved were identical for SEL1 and SEL2 levels. The highest game level reached for AS1 was 9 and 11 for AS2, respectively. Reaction speed varied from 439 to 469 ms for SEL1 and SEL2 and from 488 to 574 ms for AS1 and AS2, respectively. Correct reaction percentages varied from 96.2 to 98.5% for SEL1 and SEL2 and from 74.1 to 78% for AS1 and AS2, respectively. Incorrect reactions varied from 5.8% to 6.1% for Alert 1 and 2 (Table 2). All participants found the COPHYCON easy to use. There were no SAE in any of the 15 participants or any adverse events related to the COPHYCON device during or after game play.

### Secondary outcomes

Five participants evaluated for prefrontal cortical activity during gameplay, each produced 158 data points. Data were not normally distributed, except for the measures of both the left and right frontal lobe in the Introduction phase (Esiri and Chance, 2012), break 1 (Scafato et al., 2010), and Select S1 (Fjell and Walhovd, 2010; Table 3). There was a significant decrease of rS02 during the eight phases of cognitive game play in both the left (67.17–66.79, *p* < 0.001) and right (67.27–66.60, *p* < 0.001) prefrontal lobes (Table 3).

Small, but significant differences between left and right prefrontal rS02-values were retrieved for all game and break phases, however, not for Alert S1 game phase (*p* = 0.220) and the select S1 game phase (*p* = 0.095) Table 3). Cohens d_z_ estimated effect sizes for differences between left and right prefrontal rS02 varied between 0.2 and 0.7 (Table 3).

No significant correlations were present between the fNIRS measures and the demographic variables MOCA, age, computer use, and education. There was a trend toward significance (*p* = 0.07) between the outcome Select S1 game phase and MOCA (*r* = −0.48), explaining 23% of the variance between these measures, indicating an increased activity of the right-prefrontal cortex during exergaming in participants with MOCA scores equal or lower than 28 points. Conversely, there was no correlation between the MOCA and the fNIRS measures of the left prefrontal cortex.

A linear regression analysis revealed a significant decrease for the left ventral lobe (after Bonferroni correction Deary et al., 2009) for participants 1 and 4 during AS1 and for participant 5 during AS2. For the right ventral lobe, a significant decrease was shown for participants 1, 2, and 4 during AS1 and in participant 5 during AS2. No further significant changes, neither a decrease nor an increase could be retrieved for rS02% during exergame-play (Table 4).

**Figure 3:**
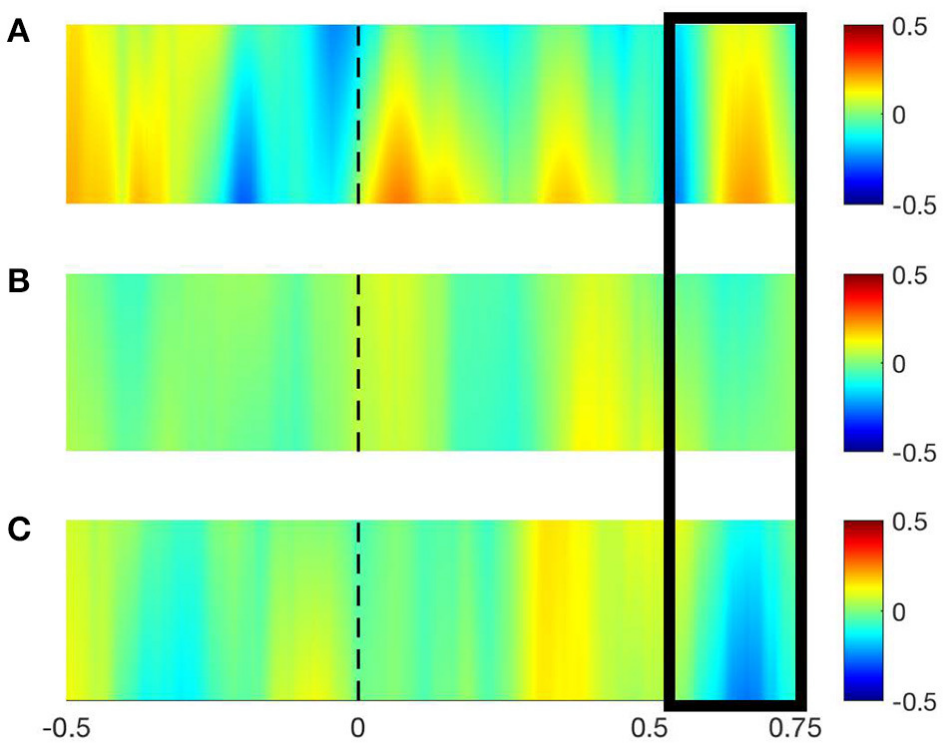
EEG time-Frequency plots (TFRs) for the game level AS1 **(A)**, AS2 **(B)**, and SEL2 **(C)** representing the evoked (log-transformed) power (range depicted in the right sided legend). TFRs are shown here for the sensor Fp1 over the left prefrontal cortex in the time interval [−0.5, 0.75 s] and in the theta frequency band [range (3.5–7.5 Hz) after applying a 2 Hz smoothing on the 5.5 Hz frequency], as the channel-frequency-time combination was significant. The dotted black line indicates the stimulus onset and the black rectangle represents the time range [~0.25, ~0.75 s] a priori selected where the significant changes in evoked power occurred over the three game levels.

The MANOVA with non-parametric permutations applied to EEG data showed a significant difference, corrected by FDR, in the Theta frequency band within the time bin ~0.5–0.75 s post stimulus onset over the left PFC sensor Fp1 [*F*_(3, 7)_ = 51.07, *p* = 0. 001], but not in the contralateral sensor Fp2 [*F*_(3, 7)_ = 1.47, *p* = 0. 75] (Figure 3). No other time channel-frequency-time bins combinations showed any significant differences (Tables 5, 6). Therefore, only a *post-hoc* analysis was performed on the significant channel-frequency-time bin of interest (Fp1–Theta—~0.5–0.75 s). The dependent *t*-test showed significant differences between the game level AS1 and SEL2 [*t*_(9)_ = 2.60, *p* = 0. 03, Cohens d_z_ estimated effect sizes = 0.82] and between SEL1 and SEL2 [*t*_(9)_ = 3.74, *p* = 0. 01, Cohens d_z_ estimated effect sizes = 1.10]. All other comparisons were non-significant.

**Table 5:** Descriptive statistic (mean and standard deviation) of all power frequency (log-transformed) x-channel, time bin, and frequency of interest for EEG measures.

**Frequency-band**	**Game**	**Latency post stimulus onset**		
		**~0–0.25 s**	**~0.25–0.5 s**	**~0.5–0.75 s**
**α** (Fp1)	AS1	0.24 ± 0.12	0.27 ± 0.17	0.35 ± 0.18
	AS2	0.27 ± 0.18	0.28 ± 0.11	0.25 ± 0.08
	SEL1	0.28 ± 0.08	0.27 ± 0.11	0.23 ± 0.08
	SEL2	0.23 ± 0.15	0.28 ± 0.15	0.29 ± 0.12
**α** (Fp2)	AS1	0.20 ± 0.12	0.07 ± 0.33	0.05 ± 0.17
	AS2	0.17 ± 0.18	0.16 ± 0.20	0.06 ± 0.13
	SEL1	0.15 ± 0.11	0.16 ± 0.12	0.14 ± 0.12
	SEL2	0.13 ± 0.13	0.16 ± 0.14	0.14 ± 0.11
**θ** (Fp1)	AS1	0.59 ± 0.33	0.55 ± 0.22	0.53 ± 0.20
	AS2	0.41 ± 0.10	0.40 ± 0.14	0.41 ± 0.19
	SE1	0.45 ± 0.12	0.47 ± 0.11	0.46 ± 0.11
	SEL2	0.42 ± 0.21	0.46 ± 0.20	0.37 ± 0.16
**θ** (Fp2)	AS1	0.55 ± 0.38	0.42 ± 0.31	0.35 ± 0.19
	AS2	0.30 ± 0.22	0.30 ± 0.15	0.34 ± 0.16
	SEL1	0.35 ± 0.13	0.34 ± 0.14	0.35 ± 0.14
	SEL2	0.33 ± 0.20	0.34 ± 0.20	0.33 ± 0.18

**Table 6:** MANOVA F Multivariate with Montecarlo permutations corrected by false discovery rate for EEG measures.

	**~0–0.25 s**		**~0.25–0.5 s**		**~0.5–0.75 s**		**EC**		**EO**	
	***F*_(3, 7)_**	***p*-value**	***F*_(3, 7)_**	***p*-value**	***F*_(3, 7)_**	***p*-value**	***F*_(3.7)_**	***p*-value**	**(F-(3.7)**	***p*-value)**
**α** (Fp1)	3.23	0.54	0.19	0.99	6.59	0.29	0.26	0.97	7.57	0.18
**α** (Fp2)	0.87	0.89	0.49	0.95	4.98	0.35	1.03	0.86	7.39	0.19
**θ** (Fp1)	8.67	0.16	7.11	0.20	**51.07**	**0.00**	1.35	0.80	8.90	0.17
**θ** (Fp2)	3.10	0.57	1.20	0.82	1.47	0.75	2.42	0.62	7.76	0.19

*EC, Eyes closed; EO, Eyes open; significant values are bold*.

## Discussion

This study tested the usability and effects of the COPHYCON in terms of (1) acceptance, adherence and attrition and safety, and (2) the effect on measures of brain functioning in a group of untrained elderly adults. The findings revealed a high level of acceptance and high adherence rates. Evaluation of subjective usefulness suggests that the participants perceived COPHYCON based exergaming as useful to train while lying supine in a hospital bed. Moreover, participants expressed a positive attitude toward the program and a moderate behavioral intention to use it. The ease-of-use was rated high and no safety issues or device related adverse events were recorded during the test phase of the COPHYCON.

Playing the games had an effect on measures of prefrontal cortex functioning as assessed with fNIRS and EEG, confirming our initial hypothesis. This is in line with Eggenberger et al. (2016), who report long-term effects of video game play on prefrontal cortex behavior and function (Eggenberger et al., 2015). Compared to our study, this work differed in methodology, as this study performed fNIRS measures with another protocol. Nonetheless, Eggenberger (Benzing et al., 2016) reported both dance and balance training activities significantly reduced left and right PFC oxygenation during acceleration of walking. In contrast to the work of Eggenberger et al., our participants were positioned in supine position and the relative Hb02 level decreased more in the right prefrontal cortex while exergaming. These differences may be explained by functional changes of the brain in elderly people. The “Hemispheric Asymmetry Reduction in Older Adults-Model (HAROLD)” (Cabeza, 2002), provides a framework for the generalization of recall-, working-, and episodic-memory tasks in the elderly brain. Younger people reportedly have a stronger activation at the unilateral side of the brain. In contrast, in elderly the activation spreads to other parts of the brain, becoming bilaterally active (Cabeza, 2002). Berlingeri et al. (2013) however, reassessed the HAROLD model, concluding that this model only captured some of the brain patterns in aging. The results of this group were more compatible to the CRUNCH (Compensatory-related utilization of neural circuits hypothesis)-model, suggesting that former neurologic patterns can be considered a special manifestation of an age related compensatory process. The *CRUNCH* model posits that additional activations, regardless of the hemispheric side, are seen in elderly participants for comparatively easier versions of the same task. However, as the task demands increases, elderly would reach a sort of plateau corresponding to the highest possible level of activation. At this point, their performance would inevitably fall due to the impossibility of meeting the additional task demands (Berlingeri et al., 2013). This might be the case in the participants in our study, as participants reached a plateau in the Select games. Another concept of “brain plasticity” is the “cognitive reserve (CR)” theory. CR postulates that individual differences in the cognitive processes or neural networks underlying task performance allow some individuals to cope better than others with brain deduction and damage (Esiri and Chance, 2012), as there are individuals with a substantial load of pathology who are able to perform with small to moderate cognitive deficits within the normal span during lifetime (Dowling et al., 2010; Esiri and Chance, 2012). Such processes reflect “functional brain and cognitive plasticity” of the deteriorating human brain (Skvarc et al., 2016). We cannot exclude that our participants had no “functional deficits,” as the only performed functional test was the MoCA.

In contrast to the decreased oxygenized Hb02 levels in four of our participants, the level of Hb02 in one participant increased in both the left and right prefrontal cortex. It is conceivable that the other participants were challenged more during gameplay. Furthermore, the increase in oxygenated Hb02 might have been caused by the electrodes being positioned to far to the temporal area, or were due to intra-individual variations of the human brain (Allen et al., 2002).

“Behavioral Intention to Use” showed that participants were motivated to use the tested games on a regular basis. The moderate acceptance rate of 65% obtained in this study seems in line with a previous study (Laver et al., 2011) that revealed skepticism about the use of the COPHYCON and cognitive games on a hospital ward. A likely explanation may be found in the good physical and mental fitness of our participants and therefore, the reduced possibility of the games to challenge the participant's cognitive potential. The cognitive games used were developed for patients with cognitive impairments and deficits. Due to the training principle of initial values, improvement in the outcomes Alertness and Selective Attention will prevail most in patients with lower initial values. In other words, those with lowest level of cognitive fitness have greatest opportunity for improvements (Ammann et al., 2014). Future studies that want to assess the effects of training in a longitudinal study design should consider this effect.

Eggenberger et al. (2016) compared an 8-week interactive cognitive-motor video game dancing intervention with a conventional balance and stretch training in elderly (Eggenberger et al., 2016). Prefrontal cortex activity was assessed during walking on a treadmill. A decrease of the HB02% at preferred and fast walking speed, as well as an asymmetry of HB02% between both the left and right prefrontal cortex during treadmill walking was revealed, with larger effects in the video game dance group (Eggenberger et al., 2016). A major difference between the study of Eggenberger et al. and ours was the dose (intensity, frequency, and time) of the performed trainings. Participants in our study trained in a single intervention, while Eggenberger et al. subjected their participants to a training program of several weeks. For future studies, the understanding of the mediating effects on the brain, while performing a physical and/or cognitive training intervention is of interest to define the dosage of such interventions. Physical activity with high cognitive engagement is likely to be more efficient than physical activity of the same intensity with low cognitive engagement (Benzing et al., 2016). Thus, the effects of exergames with higher cognitive intensities may be also effective to improve acute cognitive function in elderly with non-communicable disease (Ammann et al., 2014; Stanmore et al., 2017). This might be of interest both for health care professionals and game developers, when designing and implementing principles of exercise training in applied cognitive games (Knols et al., 2016).

In our study, EEG recordings of theta power significantly decreased in the averaged ~0.25–0.75-time interval post-stimulus onset over the left PFC sensor across the cognitive game levels, from AS1 to SEL1 and SEL2. There were no significant changes in the contralateral PFC's sensor Fp2 as well as in any of the other FOI (Table 5).

As reported by Teo et al. (2016), these results may be of interest for patients at risk for cognitive decline or with neurological disease. Rossini et al. reported “an increase” (i.e., “slowing”) of slow EEG waves (e.g., Theta frequency band) in the elderly, as progressive dysfunction and loss of synaptic contacts and neuronal abnormal apoptosis characterize the physiological aging of the brain (Rossini et al., 2013). Deterioration of Theta EEG power over the PFC was also reported in patients receiving a delirium after thoracic surgery (van der Kooi et al., 2015).

This may be of particular concern for elderly who develop surgery-related cognitive decline. Electrophysiological predictors provide a non-invasive method to preselect individuals for whom training in complex task and skill improvement outside the training task environment are more likely to be successful the higher their EEG alpha power over frontal regions is (Mathewson et al., 2012). This indicates the importance of starting rehabilitation of cognitive functioning already in early stages; e.g., when patients are confined to be immobilized: The higher their frontal alpha power is at initiation of mobilization, the better learning and skill improvements in rehabilitation will be (Mathewson et al., 2012). Moreover, Theta EEG power recorded by two sensors over the prefrontal cortex appeared to be significantly lower than those of healthy age matched controls (van der Kooi et al., 2015), and, importantly, Theta EEG power over the PFC seemed to be influenced by exergaming in older adults (Schättin et al., 2016).

These findings might have implications for the early rehabilitation of elderly with cognitive changes (and eventually for elderly who scheduled for elective surgery), as excessive synaptic-neuronal loss and/or pathologic phenomena interfere with the functional and structural plastic remodeling of the interconnectedness of brain structures.

Training with the COPHYCON implies stimulation of different multisensory mechanisms. These multisensory mechanisms relate, in turn, to brain neuroplasticity. A healthy nervous system is able responding to intrinsic and extrinsic stimuli by reorganizing its structure, function, and connections; a response ability defined as neuroplasticity (Cramer et al., 2011). In the adult nervous system alterations in central and peripheral inputs together with behavioral experience may lead to reshaping of connectivity (Ganguly and Poo, 2013). COPHYCON training offers stimuli from an artificial environment that have to be centrally processed and hence transformed into a meaningful movement. Previous research has shown that the study of cortical responses in multimodal integration sites of the brain help in understanding the process of temporally accurate motor output based on the audiovisual cues and, furthermore, it is hypothesized that changes in these relationships can be monitored to gage performance changes in motor learning (Ono et al., 2014).

In this study, a usability design without a control group was used, as the results of the fNIRS and EEG measures are considered to be preliminary work. As postulated by Whitehead et al. (2014), “The distinction between feasibility and pilot studies is still a gray area, with various definitions having been suggested by clinical trial methodology researchers” (Whitehead et al., 2014). The end-points in this study are experimental for the time being and do not include clear criteria, to proceed to a main trial. Therefore, control participants were not included, but their inclusion should be considered in future projects. In such future projects, an important research focus relates to COPHYCON training, multisensory mechanisms, and brain plasticity in individuals with surgery related immobilization. A first study has shown this to be feasible (Turon et al., 2017). As interventions aimed at maintenance of “brain activity” in the elderly has consequences on everyday life (Rossini et al., 2013), measures of independent living and mobility should also be included (Posner, 2008). To evaluate the effect of exergaming on functional outcomes, e.g., attention, should also be assessed (Spence and Driver, 1997).

## Limitations and future research

Several possible limitations of the study should be noted. First, this usability study included a user-centered interaction design with a single exergame-session. A longitudinal trend (either improvement or deterioration) in the acceptance of the COPHYCON, including the applied games or changes in the fNIRS/EEG outcomes (either improvement or deterioration) could not be determined. Second, fNIRS and EEG measures were performed with two separate devices. The results of the report of Leamy and Ward (2010) indicate that performing combined fNIRS/EEG measures should be preferred over using fNIRS or EEG devices separately, as combined FNIRS and EEG devices provide higher spatial resolution and higher temporal resolution (EEG) outcomes. Third, artifacts in fNIRS measurements are common. A difference between the measurements of the left and right sensor was reported and an asymmetry from 2 to 4% of the relative oxygen values may also occur (Casati et al., 2006). To date, there is limited guidance for clinicians to compare the accuracy of fNIRS measures, due to the possibility of the variability in methodologies, bias in patient selection, and among fNIRS devices itself. The device used in this study was able to measure the absorbed light by the electrodes 10 times per second. An average for rS02% of 50 data points was calculated, meaning that a value was shown on the fNIRS-screen every 5 s (Thavasothy et al., 2002). This procedure was not problematic in this study design, as we observed rS02%-values over 80 min; however, if the aim had been to detect fast changes in rS02 on events during cognitive gameplay, an evaluation of the efficacy of the stimuli in the games would not have been possible. Therefore, the measures and results of the INVOS 5100c device in this study cannot be compared directly with other fNIRS measures (Hyttel-Sorensen et al., 2014; Seiyama et al., 2016). Fourth, rS02% of the prefrontal cortex. Tachibana et al. (2011) performed Hb02 measures of the superior parietal lobe and superior temporal gyrus in participants performing a dance game, in which the oxygen values increased, as the game advanced in difficulty. This is relevant, as differences in the temporal relationships of the parietal lobes and the superior temporal gyrus may reflect the functional roles of the mean oxygenized hemoglobin concentration levels while performing video training. Further studies including fNIRS measures with several videogames as indicator for motor learning should therefore also measure the temporal areas, if possible. Fifth, the only neuropsychological test which was performed in advance of the exergame training was the MoCA. However, before the participants began the COGNIPLUS training, the software of the CogniPlus system demanded an initial stage-test, in which the performance level of the participant could be determined. After this stage test, the software ran a higher or lower level depending on the errors and the adequate responses, as well as to the response time of the required task during the game, e.g., a good response to a car (suddenly) crossing or not crossing the street. If the performance of a participant appeared to be better than the game level, then the level increased with one stage. Contrary, if the performance decreased, the software automatically decreased the stage to a lower level. Last, our participants were ambulatory community dwelling elderly in good mental and physical health. Their preferences for computer games and device use in rehabilitation may be different to those, whose health state depends on rehabilitation, e.g., long stay patients at risk for cognitive and functional deterioration. This might be challenging as alertness, attention and perception of these patients may vary and the time span of concentration may be short.

This study featured a rather small sample of 15 healthy elderly, which resulted in limited statistical power for fNIRS and EEG analysis and, thus, the validity of using elderly persons to test the usability may be questioned. However, the primary focus of this study was on usability, where a sample of five participants is sufficient for detecting 80% of the usability problems (Lewis, 1994). Further studies should determine the effect of studies with adequate samples.

## Conclusions

Usability studies provide invaluable results for information technology developers and rehabilitation specialists who wish to use innovative systems. Findings of such studies are relevant to the development of new emerging virtual reality-based rehabilitation methods that try to enhance prefrontal cortical involvement in elderly. This study demonstrates that the game-based intervention was perceived as usable and had a positive effect on prefrontal cortical involvement. Further testing of the device in a clinical surrounding is warranted.

## Availability of data

The dataset analyzed in the current study is available from the corresponding author(s) on reasonable request.

## Author contributions

RK: conceived the methodology and carried out quality assessment, data analysis, and manuscript writing; JS: participated in methodology conception, quality assessment, and manuscript writing; DDB: carried out data collection, quality assessment, data analysis, and manuscript writing; FG: was technical advisor for the EEG technology, carried out EEG data analysis, and participated in manuscript writing; MW: was technical advisor for the fNIRS technology and participated in manuscript writing; BK: carried out fNIRS data analysis and manuscript writing; DB: was responsible for the quality assessment and manuscript writing; EdB: supervised progress, helped with methodology conception, manuscript writing, and critical revision for scientific content; All authors read and approved the final manuscript.

### Conflict of interest statement

The authors declare that the research was conducted in the absence of any commercial or financial relationships that could be construed as a potential conflict of interest. The reviewer LL and handling Editor declared their shared affiliation.

## Footnotes

^1^L. W. *ACSM Information on Exergaming*. 2013.

^2^*Oxygenation* (2008). Available online at: http://www.bbl.unc.edu/Oxygenation.htm

## Abbreviations

AE: adverse event
BMI: bodymass index
COPHYCON: patient bedside cognitive game console
EEG: electroencephalography
EMC: electromagnetic comparability
MRI: functional magnetic resonance imagining
fNIRS: functional near infrared spectroscopy
Hb02: oxygenized hemoglobin
Hz: hertz
ICA: independent component analysis
ICU: intensive care unit
LCD: liquid crystal display
mbits: megabits
MOCA: Montreal cognitive assessment
ms: milliseconds
PFC: prefrontal cortex
RP: relative power
PSD: power spectral density
PSI: Paul Scherrer Institute
rS02: intracranial regional hemoglobin oxygen saturation
SAE: severe adverse event
SD: standard deviation
TAM: technical acceptance model
μV: microvolt.
